# Contrasting Population and Diet Influences on Gut Length of an Omnivorous Tropical Fish, the Trinidadian Guppy (*Poecilia reticulata*)

**DOI:** 10.1371/journal.pone.0136079

**Published:** 2015-09-11

**Authors:** Eugenia Zandonà, Sonya K. Auer, Susan S. Kilham, David N. Reznick

**Affiliations:** 1 Department of Biodiversity, Earth and Environmental Science, Drexel University, Philadelphia, Pennsylvania, United States of America; 2 Department of Biology, University of California, Riverside, California, United States of America; University of Padova, ITALY

## Abstract

Phenotypic plasticity is advantageous for organisms that live in variable environments. The digestive system is particularly plastic, responding to changes in diet. Gut length is the result of a trade-off between maximum nutrient absorption and minimum cost for its maintenance and it can be influenced by diet and by evolutionary history. We assessed variation in gut length of Trinidadian guppies (*Poecilia reticulata*) as a function of diet, season, ontogeny, and local adaptation. Populations of guppies adapted to different predation levels have evolved different life history traits and have different diets. We sampled guppies from sites with low (LP) and high predation (HP) pressure in the Aripo and Guanapo Rivers in Trinidad. We collected fish during both the dry and wet season and assessed their diet and gut length. During the dry season, guppies from HP sites fed mostly on invertebrates, while guppies in the LP sites fed mainly on detritus. During the wet season, the diet of LP and HP populations became very similar. We did not find strong evidence of an ontogenetic diet shift. Gut length was negatively correlated with the proportion of invertebrates in diet across fish from all sites, supporting the hypothesis that guppy digestive systems adapt in length to changes in diet. Population of origin also had an effect on gut length, as HP and LP fish maintained different gut lengths even in the wet season, when their diets were very similar and individuals in both types of populations fed mostly on detritus. Thus, both environment and population of origin influenced guppies gut length, but population of origin seemed to have a stronger effect. Our study also showed that, even in omnivorous fish, gut length adapted to different diets, being more evident when the magnitude of difference between animal and plant material in the diet was very large.

## Introduction

Phenotypic plasticity in behavior, morphology, and physiology is advantageous for organisms that live in variable environments, provided that cues of environmental change are reliable and the costs of plasticity are not too great [1]. When environments fluctuate infrequently, phenotypic plasticity is often irreversible [2]. However, reversible plasticity is generally favored when environmental changes take place throughout an individual’s lifetime, thus allowing the organism to switch from one phenotype to another as the environment changes [2].

A change in digestive tissues in response to a change in diet quality is a classic example of reversible phenotypic plasticity or phenotypic flexibility [3–8]. Many bird and lizard species change their gut size seasonally as a consequence of fluctuating resource quantity and quality (reviewed in [7,9]). Eurasian perch exhibit adaptive plasticity in their gut length as a consequence of changes in diet quality, developing longer guts when exposed to poorer quality food types both in the lab and in the wild [6]. However, animals cannot maintain a unique digestive system that is simultaneously adapted for every type of diet because different food types are absorbed through different biochemical pathways or have different processing times [10–12]. For instance, organisms need longer guts to digest lower quality food (e.g. fiber-rich) than more easily digestible food (e.g. protein-rich) [6,13,14]. Longer guts have higher surface area and allow a longer retention time of the food, consequently enhancing nutrient absorption [15]. Additionally, digestive tissues are very expensive to maintain, so it is critical to adjust them to an optimal energy intake/maintenance balance [10,15]. Gut flexibility is therefore of paramount importance for those animals that feed on a wide array of food types or that live in environments with high temporal and spatial variation in resources [10]. Such animals can display high plasticity in their digestive systems because they often shift to different types of food that have different digestive requirements (e.g. animal vs. plant food) [4,7,16]. However, not much is known about how gut length of omnivorous animals responds to intraspecific variation in diet, other than ontogenetic shift [14].

While gut length can change in response to fluctuations in diet quality, it can also be affected by evolved differences in diet as well as phylogenetic history [13,17,18]. For example, a study on Lake Tanganika cichlids showed that both phylogenetic history and diet explained species differences in intestine length, even if diet had a much stronger predictive effect [13]. Interspecific differences in intestinal length of terapontid fish from Australia also have a phylogenetic component in the ontogenetic developmental mechanisms, and were also strongly correlated with species differences in diet [17]. Both evolutionary history and plasticity in diet quality can therefore drive variation in gut length, but have generally been studied separately so their relative effects are not well known. Dramatic changes in diet, for example those associated with seasonality or fluctuations in food quality and quantity, may swamp out or even exaggerate effects caused by evolutionary differences. However, limited attention has been devoted to gut length plasticity in omnivorous organisms, including considerations of the extent to which gut morphology is affected by natural temporal and spatial variation in diet or by evolutionary effects (but see [19]).

In this study, we investigate the relative effects of population differences and seasonal variation in diet quality on gut length in a tropical omnivorous fish, the Trinidadian guppy (*Poecilia reticulata*). Guppies inhabit freshwater streams characterized by gradients in predation pressure, population density, and productivity. Higher order stream reaches are generally larger, have higher productivity and a more diverse predator community relative to smaller headwater tributaries, where barrier waterfalls prevent the upstream movement of all but one guppy predator [20]. These differences in predation risk and productivity among stream reaches have led to evolutionary differences in diverse traits among guppy populations, including life history [21,22], morphology [23,24], and behavior [25,26].

We have previously demonstrated that guppies from downstream high predation (HP) and upstream low predation (LP) populations also have different diets. Individuals from both LP and HP populations feed on invertebrates, algae and detritus [27–29]. However, HP populations are more selective, feeding mostly on invertebrates, while LP populations are more omnivorous and less selective [28]. These differences in preference are maintained even under common garden conditions [29], suggesting genetic divergence in diet among populations. Streams inhabited by guppies often undergo rapid shifts in the quantity and quality of available food resources due to changes in precipitation patterns associated with distinct wet and dry seasons [30–32]. During the wet season, flooding and associated scouring event frequency increases, thereby reducing benthic invertebrate abundance [33,34]. In the wet season, fish densities decline and the competitive or predatory interactions might weaken [35]. These temporal changes in food resource availability might force guppies to rely more heavily on lower quality food sources during the wet season, thereby leading to concomitant changes in their gut length.

Here, we first compared diet and gut length between HP and LP guppy populations from two different river systems to assess whether population differences in diet, established by our previous work, have led to corresponding differences in gut length. We then compared diet and gut length between the wet and dry season in a single pair of LP and HP populations to determine whether guppies exhibit seasonal variation in their diet and corresponding gut length. These two approaches allowed us to contrast the effects of population divergence versus plasticity on differences in both diet and gut length, because we can ask if the differences between the LP and HP populations persist independently of environmental changes. Finally, we investigated the existence of an ontogenetic diet shift in guppies. To our knowledge, this is the first study that aims to make a direct comparison of the effects of evolutionary mechanisms and phenotypic plasticity on diet and gut length in natural populations of an omnivorous fish.

## Materials and Methods

We conducted this study in the Aripo and Guanapo Rivers on the southern slope of the Northern Range Mountains of Trinidad. In each river, guppies were collected from two sites, one upstream low predation (LP) and one downstream high predation (HP) site, for a total of 4 sites: Aripo HP, Aripo LP, Guanapo HP, Guanapo LP. We collected fish in July 2006 (wet season, Aripo River only) and March 2007 (dry season, Aripo and Guanapo Rivers) on individual dates for each site. In each site, we collected guppies from three pools and within each pool, from areas with different stream velocity (low, medium, and high) to ensure sampling of most of the microhabitats guppies use in the stream. Fish were collected with hand nets and euthanized immediately with an overdose of the anesthetic MS-222. Guppies were then measured for standard length, weighed, and guts were removed. Guts and fish were preserved in 5% formalin solution. Guts were measured for length and dissected to determine diet content.

### Diet analysis

A total of 41 guppies (21 for HP and 20 for LP) in the dry season and 54 guppies (26 in HP and 28 in LP) in the wet season from the Aripo River, including males, females and juveniles, were analyzed for diet content using protocols established during our previous research on guppy diet [28]. Briefly, only the stomach and a small part of the foregut–where it turns 180 degrees–were used for the analysis, because the content in the hindgut was too digested. The gut contents were placed on a gridded slide and invertebrates were identified to the lowest taxonomic level using a dissecting scope [36,37]. Then the material was distributed evenly across the slide and ten squares (out of 64) were chosen randomly for quantification of the gut contents under a compound microscope. For each square, the area covered by invertebrates and detritus was estimated, while diatoms and filamentous algae were counted because they are too small to calculate their area coverage. An average size for diatoms and one for filamentous algae was subsequently assigned to calculate the area they occupied in the 10 squares. The area taken by each food category was then calculated for the entire slide. Occasional or rare items found in the gut (e.g. plant material, inorganic material, and other algae) were not included in the analysis. For the Guanapo River, we used the data from [28].

### Gut length measurements

Gut length was measured in a different set of Aripo guppies from the dry (39 for HP, 41 for LP) and the wet season (23 for HP, 21 for LP) and from Guanapo guppies just from the dry season (21 for HP and 21 for LP). Individual guts were placed in a petri dish and cut into 2–3 parts, as the intestines can be very convoluted. In this way we could measure every part without stretching the gut, which could bias the total length measurement. Individual guts were photographed with a digital camera connected to a Leica dissecting microscope and then measured using the software ImageJ. Three different measurements were taken for each gut. These measurements were highly repeatable (intraclass correlation coefficient: 0.999, P<0.001), so we used the average of them in our analyses of gut length.

### Statistical analysis

We used a multivariate analysis of covariance (MANCOVA) to test for population of origin (HP versus LP) and seasonal effects (dry versus wet) on the proportion of food items (invertebrates and detritus) in the guppy diet from the Aripo River. We used fish length as a covariate, because fish often switch their diet with age/size. We did not include the proportion of algae as a dependent variable into the MANCOVA because it violated the assumptions of equality of variances and normal distribution of the residuals. The MANCOVA showed a significant season x fish length interaction, so we further explored the causes of this interaction. We first ran univariate ANCOVAs for each of the 2 dependent variables, then linear regressions, and finally a Johnson-Neyman procedure, which tested the effect of season (fixed factor) at different values of fish length (covariate) in order to identify the value of the covariate where the interaction becomes significant [38].

To test for differences in gut length between groups in the Aripo River guppies, we employed a two-way ANCOVA, with fish standard length as a covariate because of the allometric relation between fish length and gut length. In the model, population of origin (HP or LP), season (dry or wet), their interaction, and the interaction between fish length and population were the main effects. We did not include the interaction between fish length and season because it was not significant. Considering that guppies from HP and LP populations did not overlap extensively in their body sizes in the wet season sample, we also ran the same type of ANCOVA but only including guppies between 14 and 20 mm in size. This size range, indeed, represents the highest overlapping sample size across all sites and seasons. In this analysis we included population of origin (HP or LP), season (dry or wet), their interaction, and fish length as main effects, but we did not include in the model the interactions between fish length and population of origin and between fish length and season because they were not significant. Additionally, we ran individual ANCOVAs for each population (Aripo HP and Aripo LP) to test for differences in gut length between seasons, where fish length was the covariate. Because the interaction between fish length and season was not significant, we did not include it in the model.

For the Guanapo fish, we performed a one-way ANCOVA, with fish length as our covariate, to test for differences in gut length between low predation (LP) and high predation (HP) guppies. Because there was no significant interaction between population of origin and fish length, we removed this effect from the model and left only population of origin as a fixed factor.

Finally, we ran a linear regression analysis to examine the relationship between relative gut length and proportion of invertebrates in the diet in guppies from all datasets together (Aripo dry and wet season, Guanapo dry season). In the regression analysis we only included fish between 14 and 20 mm standard length, so we could minimize the effects of the allometric relationship between gut length and fish length (see also [39,40]). Guppy body length ranges from approximately 8 to 25 mm and they are generally sexually mature above 12 mm [22]. The relative gut length was calculated as the gut length divided by the fish length. The use of this metric can be controversial when used to compare individuals of different sizes [39,40], but we minimized this problem by using fish of the same size class across all sites. Because we did not have both gut length and diet measurements for each individual fish, an average value was assigned for the proportion of invertebrates for each population and season (Aripo HP and LP for both dry and wet season; Guanapo HP and LP for only the dry season), which was the estimated marginal mean obtained from the diet analysis (the Guanapo River means are from [28]).

Gut length, fish length, and relative gut length measurements were log transformed while the proportion of food items values was arcsin square root transformed to meet the assumption of normality. All levels of significance were set at 0.05 and statistical analyses were performed using SPSS Statistics 19.0.0 (SPSS inc.).

### Ethical statement

This study was carried out in strict accordance with the recommendations in the Guide for the Care and Use of Laboratory Animals of the National Institutes of Health. Fish were collected and handled with the approval of the University of California Riverside IACUC AUP (no. A-20080008). Fish collection and export was approved by Ministry of Agriculture, Land and Marine Resources, Republic of Trinidad and Tobago, conforming to their legislation. All fish were euthanized with an overdose of MS-222 to minimize suffering.

## Results

### Seasonality in diet

**Fig 1:**
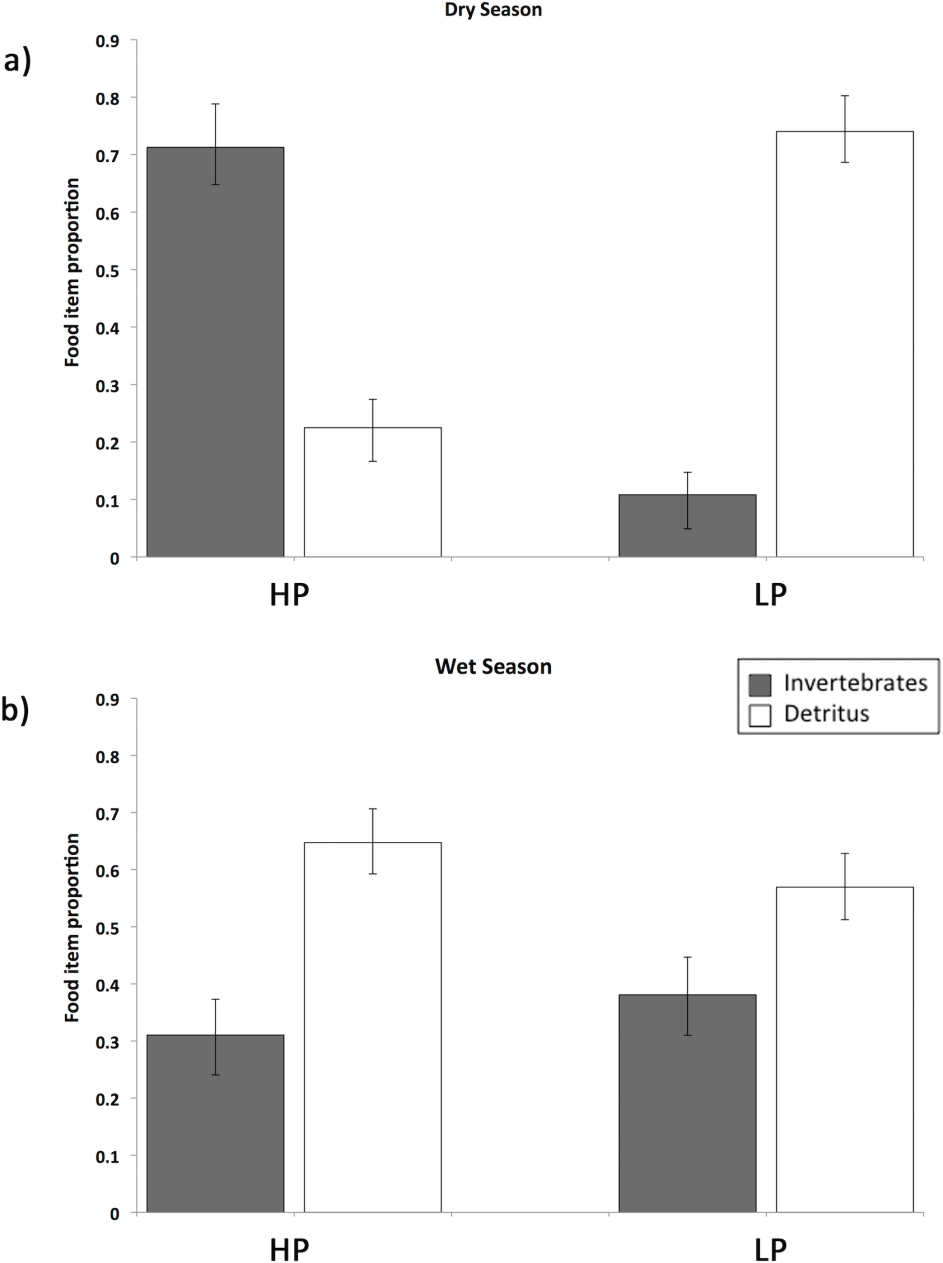
Season effect on the diet of HP and LP guppies from the Aripo River. Percent diet composition is shown for the dry (a) and the wet (b) seasons. Data showed here represent the estimated marginal means calculated by the MANCOVA on arcsin transformed data. Estimated marginal means and standard errors have been back-transformed for the graphical representation. Food categories analyzed are invertebrates, in dark grey and amorphous detritus. LP: low predation; HP: high predation. Bars represent ±1 SE.

**Fig 2:**
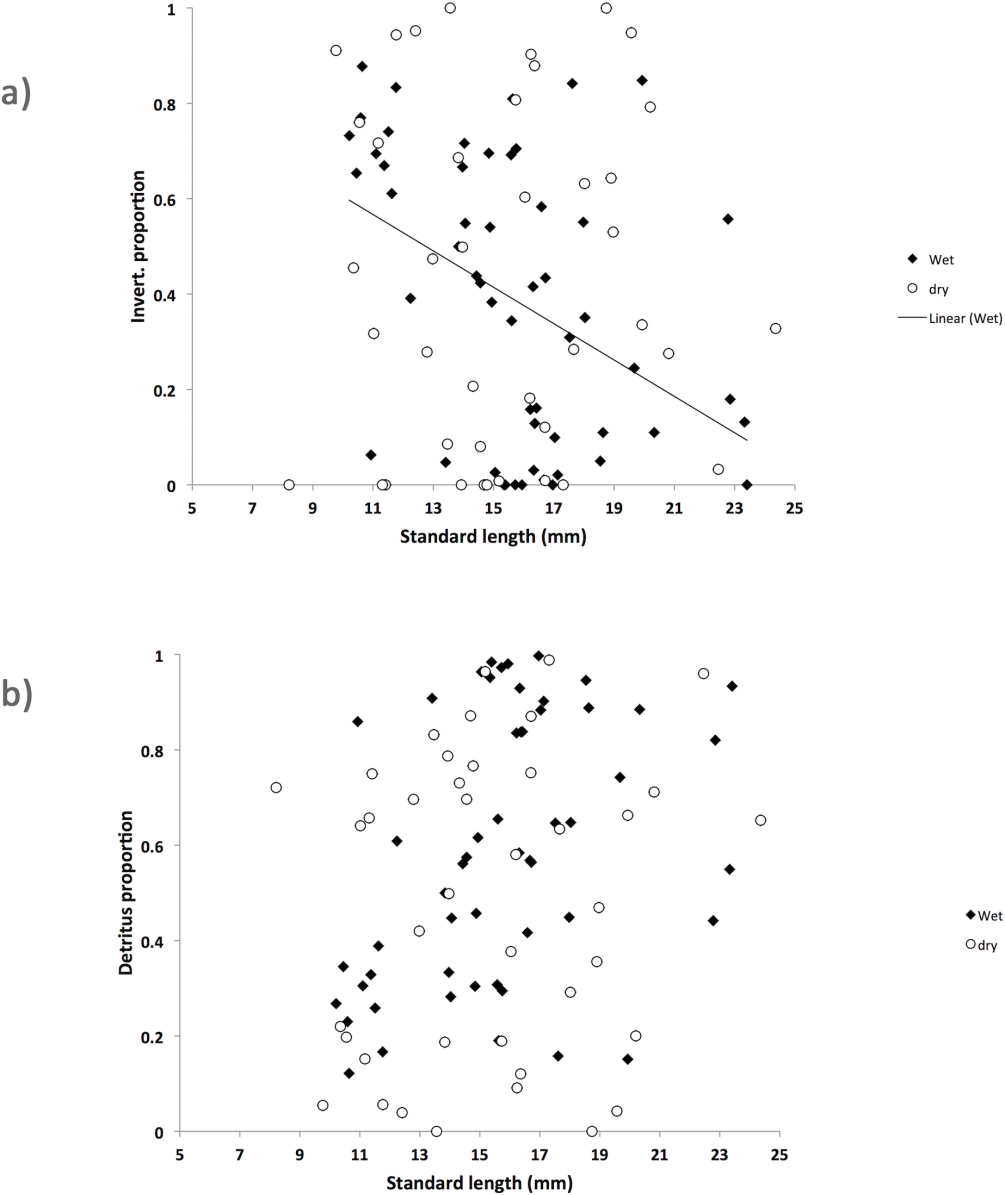
Correlation between guppy standard length and a) proportion of invertebrates and b) proportion of detritus in guppies diet from the Aripo River. Wet season (filled diamonds) and dry season (open circles) fish are shown. Regression line shows the only significant relationship, which is between proportion of invertebrates in diet and guppy length during the wet season.

Guppies from the Aripo River change their diet with season. While in the dry season there were significant differences between HP and LP fish diets, in the wet season these differences disappear and HP and LP fish have essentially the same diet (Fig 1). The MANCOVA showed a significant effect of population of origin (F_2,88_ = 7.543, P = 0.001), season (F_2,88_ = 10.168, P<0.001), and of the interaction between season and population of origin (F_2,88_ = 12.294, P<0.001). Fish length also had a significant effect (F_2,88_ = 8.085, P = 0.001), as well as the interaction between fish length and season (F_2,88_ = 7.572, P = 0.001). This heterogeneity of slopes was generated by the different correlation between proportion of invertebrates and fish length between the dry and wet season (univariate ANOVA: F_1,89_ = 7.146, P = 0.009) (Table 1 and Fig 2A), but not for the proportion of detritus and fish length between seasons (univariate ANOVA: F_1,89_ = 2.344, P = 0.129) (Table 1 and Fig 2B). We therefore ran two regression analyses to further examine the relation between the proportion of invertebrates and fish length for each season. In the wet season, there was a significant (F_1,53_ = 9.464, P = 0.03; y = -0.045x + 1.33, r^2^ = 0.15) negative relationship between the proportion of invertebrates and fish length (Fig 2) while there was no significant relationship between consumed invertebrates and fish length in the dry season (F_1,40_ = 0.100, P = 0.753; y = 0.007x + 0.57, r^2^ = 0.03) (Fig 2). We ran a Johnson-Neyman procedure for the proportion of invertebrates, where fish length was the covariate and season the fixed factor, which showed that the season x fish length interaction became significant when guppies where bigger than 20.25mm (P = 0.05).

**Table 1:** Univariate ANCOVA results on the effects of population of origin and season on the proportion of invertebrates and detritus in the diets of guppies from the Aripo River in Trinidad. SS = sums of squares; df = degrees of freedom; MS = mean squares. All F-ratios are based on type-III sums of squares.

Univariate ANCOVA					
Source	SS	df	MS	F-ratio	P-value
*Invertebrates*					
Population of origin	1.93	1	1.93	15.26	<0.001
Season	0.79	1	0.80	6.26	0.014
Fish Length	0.40	1	0.40	3.15	0.079
Population of origin*Season	3.01	1	3.01	23.88	<0.001
Season*Fish Length	0.90	1	0.90	7.15	0.009
Error	11.23	89	0.13		
*Detritus*					
Population of origin	1.16	1	1.16	13.45	<0.001
Season	0.10	1	0.10	1.11	0.295
Fish Length	0.73	1	0.73	8.49	0.005
Population of origin*Season	2.10	1	2.10	24.31	<0.001
Season*Fish Length	0.20	1	0.20	2.34	0.13
Error	7.71	89	0.09		

The diet differences between LP and HP guppies in the Guanapo River (Table 2) do not have the same magnitude as in the Aripo River (Fig 1A). The guts of HP guppies from both rivers contained a similar percentage of invertebrates (70% for Aripo, 65% for Guanapo). The LP guppies from the Guanapo River had consumed 34% invertebrates, as compared to only 10% for the LP guppies from the Aripo River (data from [28]).

**Table 2:** Diet composition of Guanapo River guppies from high predation (HP; N = 21) and low predation (LP; N = 21) sites during the dry season (data from [28]). Proportions of the 3 food items are estimated marginal means (±SE) calculated by the MANCOVA (population of origin as the fixed effect and fish length as a covariate); data reported have been back-transformed.

Population of origin	Food item	Proportion
HP	Invertebrate	0.65 (0.06)
HP	Detritus	0.32 (0.06)
HP	Algae	0.01 (0.00)
LP	Invertebrate	0.34 (0.08)
LP	Detritus	0.49 (0.09)
LP	Algae	0.05 (0.02)

### Gut length

**Fig 3:**
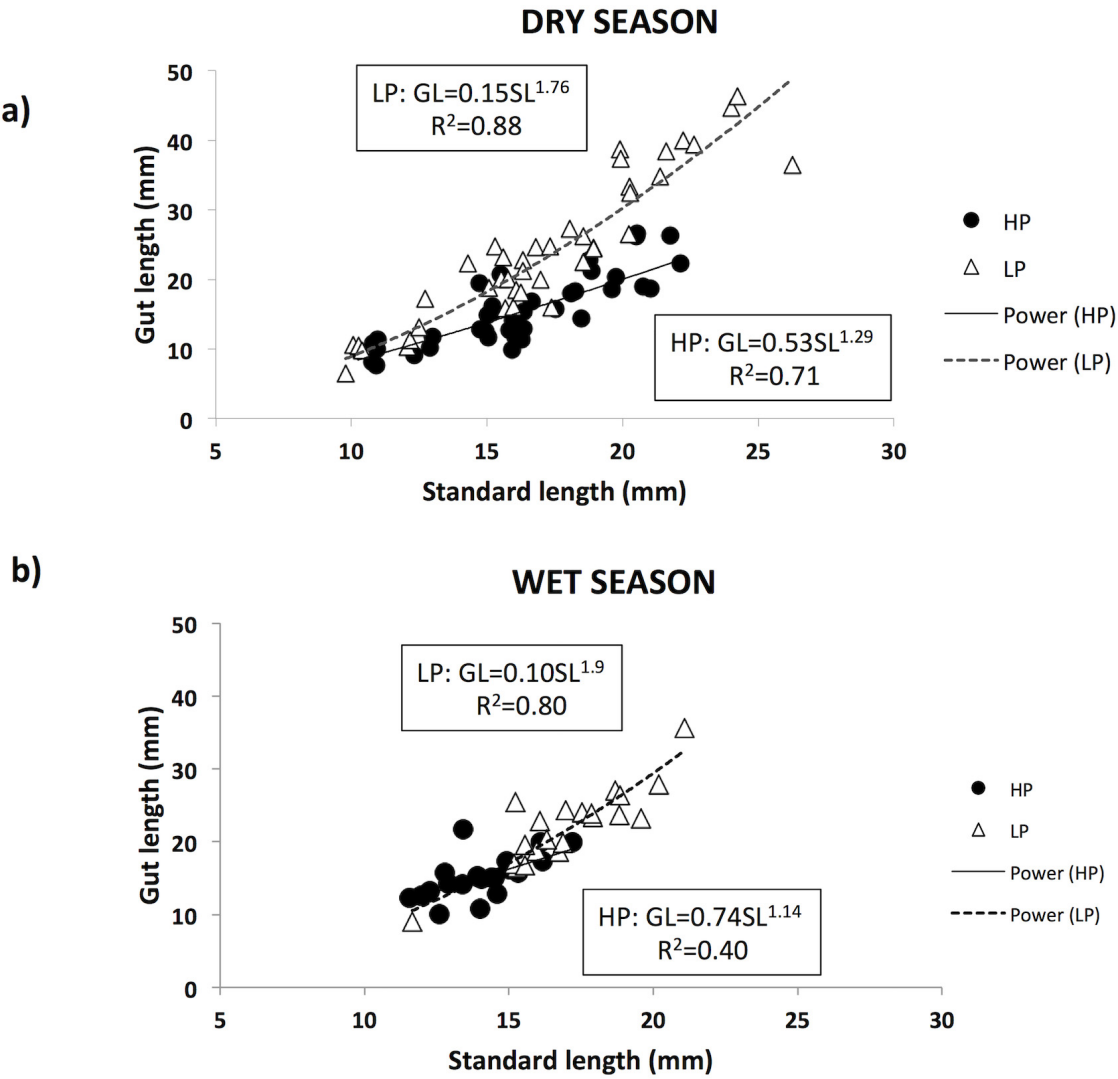
Correlation between fish standard length and guppies gut length in the Aripo River. Guppies from high predation (HP, filled circles) and low predation (LP, open triangles) populations in the dry (a) and wet (b) season are shown. Data are not transformed and equations and R^2^ values are calculated on non-transformed data. Trendlines are exponential.

For the Aripo River guppies, the two-way ANCOVA confirmed the allometric relationship between gut length and fish length (fish length effect: F_1,118_ = 423.510, P<0.001). It also showed a significant effect of population of origin (F_1,118_ = 8.654, P = 0.004) and marginally significant effect of season (F_1,118_ = 3.792, P = 0.054) on gut length in guppies. However, there were also significant interactions between population of origin and season (F_1,118_ = 12.919, P<0.001), and between population of origin and fish length (F_1,118_ = 11.565, P = 0.001). Because of the heterogeneity of slopes due to the significant interaction between population of origin and fish length, we need to be careful interpreting the results for the main effects (population of origin, season, and their interaction). For this reason, we also ran regression analyses for each population and each season to examine in more detail the relationship between gut length and fish length (Fig 3). We found that the slopes of the linear regressions were always steeper and the intercept always higher for LP guppies (dry: F_1,39_ = 278.32, P < 0.001, y = 1.76x – 0.81, r^2^ = 0.88; wet: F_1,19_ = 74.31, P < 0.001, y = 1.9x – 1, r^2^ = 0.80) than for HP guppies (dry: F_1,37_ = 91.68, P < 0.001, y = 1.29x – 0.38, r^2^ = 0.71; wet: F_1,21_ = 14.15, P = 0.01, y = 1.14x – 0.13, r^2^ = 0.40) both in the dry and wet season. This indicates that guppy guts from the LP site increase in size with fish length faster than fish from the HP site. The wet season samples were more limited in size range, with HP fish being mostly small individuals and LP mostly big individuals, thus reducing the overlap in size between the two sites. This limited overlap made the patterns in gut length more difficult to interpret. For instance, the regression analysis for the LP site is highly affected by the only small fish data point (size: 11.66 mm). When this individual is removed from the analysis, the equation and r^2^ value change substantially (F_1,18_ = 32.20, P < 0.001; y = 1.57x – 0.6; r^2^ = 0.64), reducing the difference in slope between LP and HP guppies. Overall, in the dry season there is an obvious difference in gut length patterns between HP and LP guppies, with LP fish having overall longer guts in particular when fish are above a certain size. In the wet season the patterns are not so clear, mostly due to the more limited size range and the small range of overlap in sizes of our samples (Fig 3).

**Fig 4:**
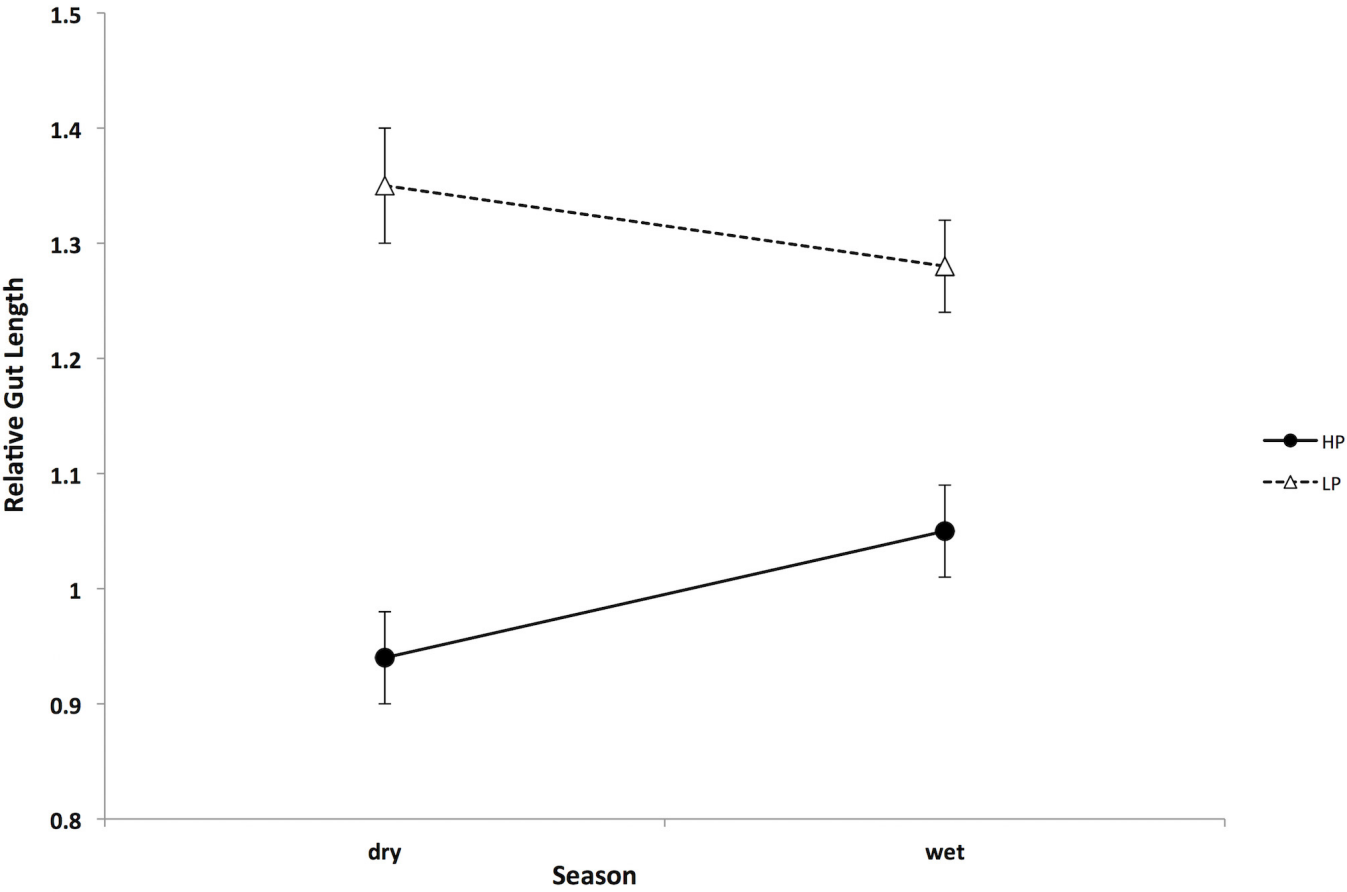
Mean guppies gut length for HP and LP guppies during the dry and the wet season in the Aripo River. Only data from guppies between 14 and 20 mm are included in this graph. Guppies from the HP (high predation) population are shown in filled circles, while guppies from the LP (low predation) population are in open triangles. Bars represent ±1 SE.

To overcome the difficulties in interpreting our Aripo River gut length data due to the heterogeneity of slopes and the limited overlap in sizes, we ran an ANCOVA including only guppies between 14 and 20 mm. This analysis still showed a significant effect of population of origin (F_1,72_ = 35.42, P<0.001), population of origin x season (F_1,72_ = 6.65, P = 0.012), and fish length (F_1,72_ = 45.49, P<0.001) on gut length. Season did not have a significant effect on gut length (F_1,72_ = 2.429, P = 0.123) of guppies from the Aripo River. While in the dry season there were big differences in gut length between guppies from the HP and LP populations, in the wet season these differences are substantially reduced. The ANCOVAs performed for each population of origin separately (again with only 14–20mm guppies included) showed that HP guppies had significantly greater gut lengths in the wet season (season effect: F_1,33_ = 5.46, P = 0.026), while in LP fish there was not a significant effect of season (season effect: F_1,38_ = 0.69, P = 0.41) (Fig 4). As for previous analysis, both ANCOVAs, for HP and LP, showed a significant effect of fish size on gut length (HP: F_1,33_ = 17.33, P = 0.02; LP: F_1,38_ = 28.39, P < 0.001).

Population of origin did not have a significant effect on gut length in the Guanapo River (F_1,77_ = 5.571, P = 0.073). The regression equations for HP and LP guppies are very similar, but LP slopes were steeper than HP ones (HP: F_1,39_ = 160.42, P < 0.001; y = 1.515x – 0.56, r^2^ = 0.80; LP: F_1,37_ = 211.30, P < 0.001; y = 1.75x – 0.88, r^2^ = 0.85), confirming the patterns found in the ANCOVA. Thus, while the ANCOVA showed no significant differences between HP and LP populations, the trends in the data are the same as in the Aripo data, which are that the HP fish consume more invertebrates and have a lower slope for the relationship between size and gut length.

**Fig 5:**
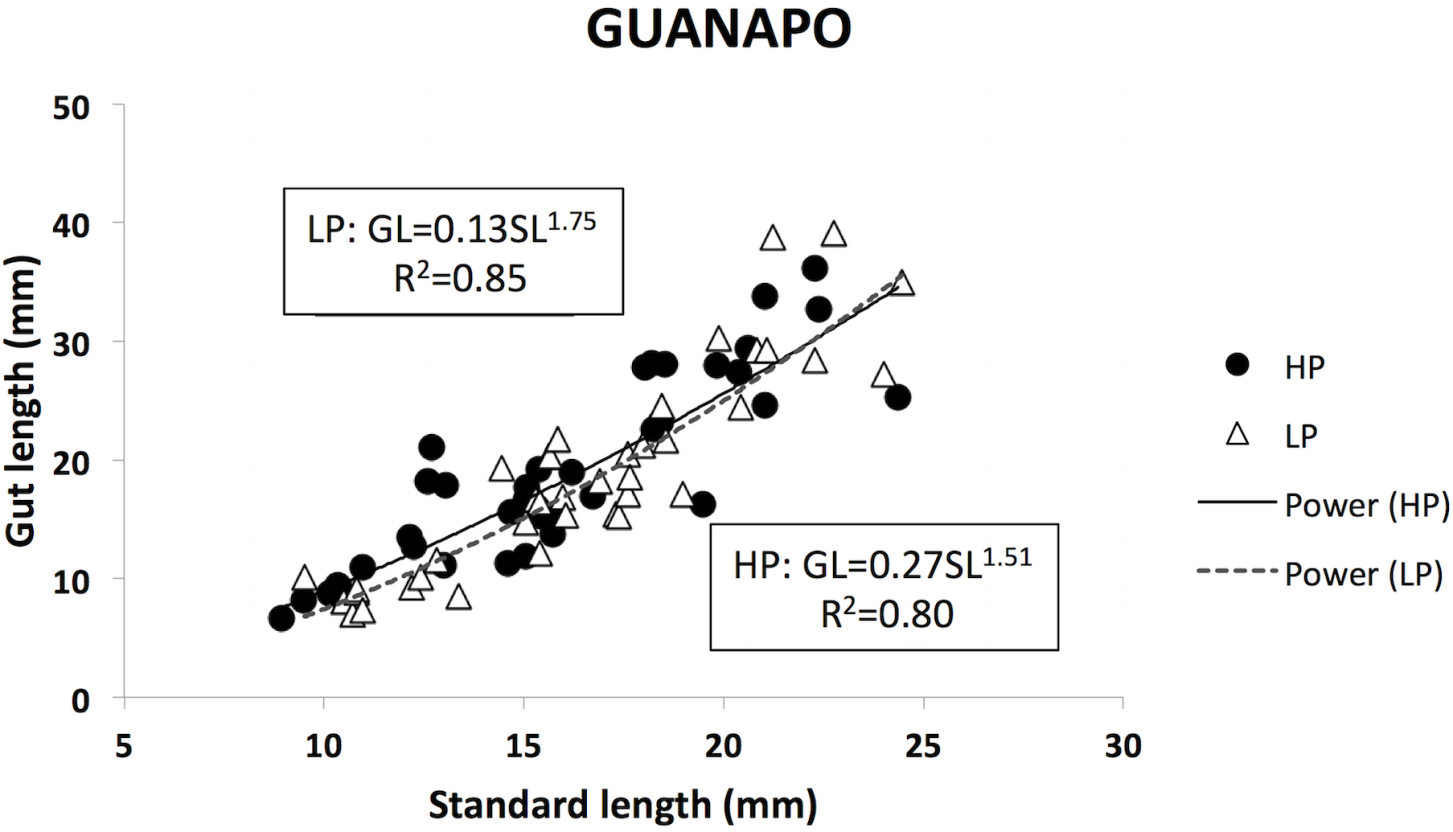
Correlation between fish standard length and guppies gut length in the Guanapo River. Fish from high predation (HP) are indicated with filled circles, and from low predation (LP) with open triangles. Data are not transformed and equations and R^2^ values are calculated on non-transformed data. Trendlines are exponential.

The allometric equations for the relationship between gut length and fish length accounted for significant variation in gut length in all six data sets, presenting high r^2^ values that ranged from 0.40–0.88 (average = 0.74). The slope of the allometric equations ranged from 1.14–1.9, indicating that guppy intestine length always increases faster than body size (Figs 3 and 5). LP guppies always exhibited steeper slopes than HP guppies.

**Fig 6:**
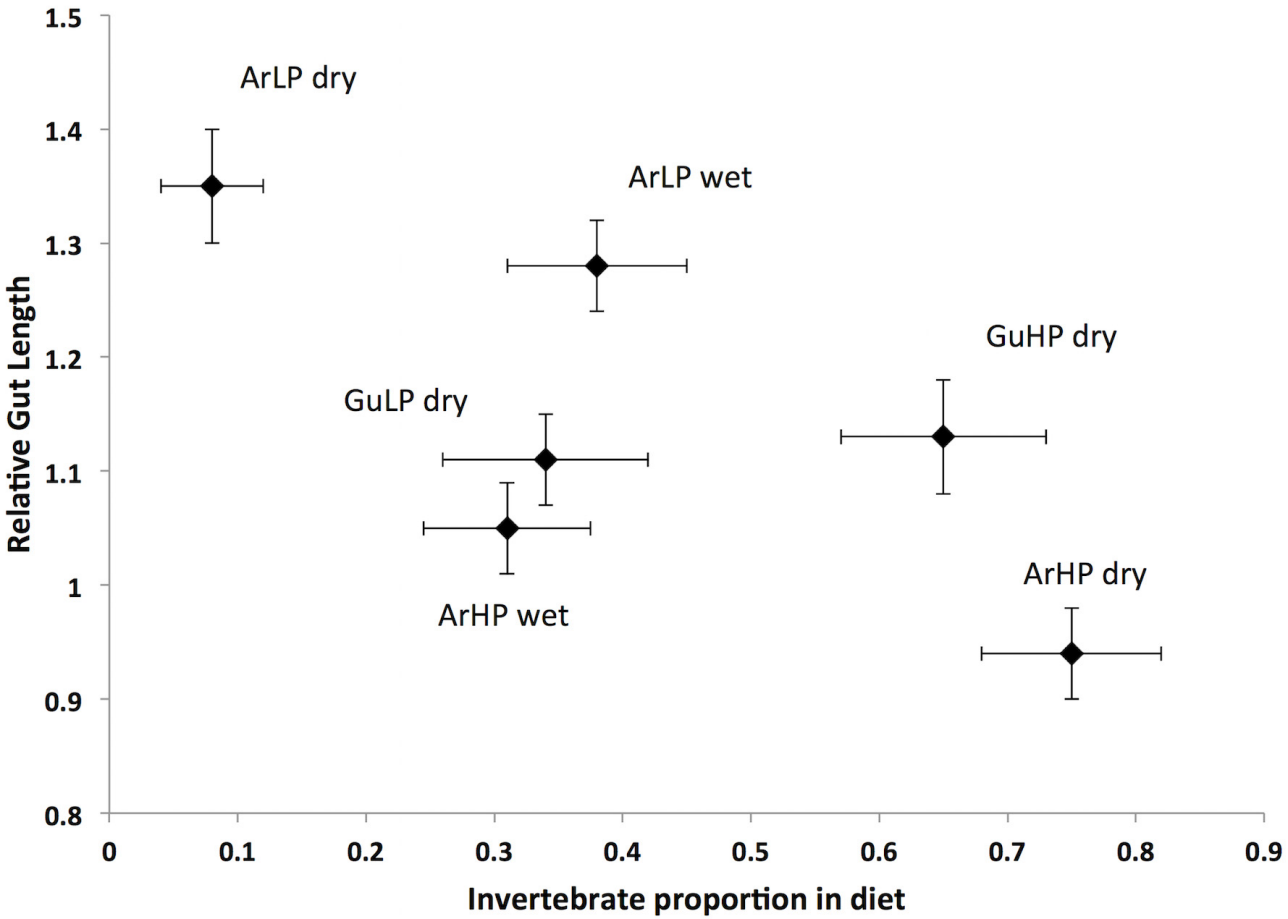
Mean proportion of invertebrates in diets vs. mean relative gut length. Only guppies between 14 and 20 mm were included. Each data point represents one site (Aripo HP and LP for both dry and wet season, and Guanapo HP and LP from the dry season). Relative gut length was calculated as the gut length divided by fish length. An average value was assigned for the proportion of invertebrates for each site, which was the estimated marginal mean obtained from the diet analysis (data for the Guanapo fish are from [28]).

We found a significant negative relationship between the proportion of invertebrates in the diet and relative gut length across all our guppy samples (F_1,117_ = 31.46, P < 0.001; y = -0.125x - 0.009; r^2^ = 0.21). As the proportion of invertebrates in the diet decreased (indicating a herbivory increase and thus an overall lower quality diet), we detected a progressive increase in the relative gut length of guppies (Fig 6).

## Discussion

The results of this study showed that both local adaptation and season had an effect on the diet of an omnivorous fish, influencing the relative proportion of vegetal and animal matter in its diet. We also showed that guppy gut length responded to these differences in diet, and that it was negatively correlated with the proportion of invertebrates found in their diet. Gut length in guppies was strongly influenced by local adaptation (high predation vs. low predation) and by its interaction with environmental variability (season). However, the effect of local adaptation appeared to be stronger, as the differences in gut morphology, even if smaller in magnitude, were maintained between HP and LP guppies, in spite of a change in diet with season. In the dry season, the two guppy populations differ in their diet and consequently in their gut length (HP guppies are more carnivorous and have shorter guts compared to LP guppies). In the wet season, HP and LP guppies change their diet, becoming very similar because HP guppies consume more detritus and fewer invertebrates. Their gut length increased in association with this change but HP guppies still had proportionally shorter guts than LP guppies (Fig 4). Most likely, gut length responded to diet differences mainly when the magnitude of diet change was big, as seen for HP guppies. Finally, we found very limited indication of an ontogenetic shift in diet in guppies, where just in the wet season there was a decrease in invertebrates in the diet with increasing size. There was a positive allometric relationship between gut length and fish length, with exponential curves always higher than 1 for all sites and seasons, which is a common pattern among many fish species [39].

### Seasonality in diets

The diets of high and low predation guppies from the Aripo River differed significantly during the dry season (Fig 1 and Table 2). During the dry season, LP guppies fed more consistently on low quality food–detritus–and showed relatively longer guts compared to HP fish, which instead had a higher quality diet (mostly invertebrates) and had comparatively shorter guts. In the wet season, guppies from both populations switched their diet: in LP sites they increased the proportion of invertebrates in their diet, while HP guppies decreased it and ate more detritus. As a result, in the wet season the differences in diet between high and low predation guppies became negligible.

We recognize two possible causes of HP guppies feeding on a higher proportion of invertebrates during the dry season. One is that invertebrates may be more abundant [28]. The second is an indirect consequence of predators, which is that guppies are less abundant [20]. The lower population density of guppies is associated with a higher individual growth rate, which we assume is associated with higher per capita food availability [20]. Conversely, guppies from LP environments, where they are released from predation, are found at higher population densities and have lower growth rates. HP guppies may have a higher quality diet during the dry season as a consequence of their lower population density and higher invertebrate abundance.

There are several possible explanations for why guppies switch their diet between seasons. During the wet season the density of guppies decreases due to both the increased volume of water in the streams and the reduction in population size caused by a reduction in the rate of reproduction [41]. We might thus expect that in the wet season the per capita resources available increase in LP sites and intraspecific competition decreases. As a response, guppies could increase the amount of higher quality food (invertebrates) in their diet, especially in those streams where intraspecific competition was higher during the dry season and the access to high quality resources more limited. This would explain the higher amount of invertebrates in the diet of LP guppies in the wet season compared to the dry season, which is the site where we see higher guppy density [42,43] and thus stronger intraspecific competition.

However, in the wet season, especially after heavy rain episodes, the amount of resources, such as periphyton and benthic invertebrates, drops off [33,44,45], thus reducing food sources available to the guppies. Downstream high predation sites are generally wider, while upstream low predation sites are narrower and have higher canopy cover [20], a distinction that also characterizes our two Aripo sites. LP sites might recover much faster from heavy rain events than HP, where the scouring of resources may be more severe because they receive the water from all upstream tributaries. The guppy populations in downstream HP sites might thus be more heavily affected by the increasing precipitation during the wet season, suffer a greater decline in food availability and hence be forced to shift to a lower quality diet. However, our dataset is limited to only one pair of HP-LP populations with only one year of dry-wet season comparison. We thus need more replicates to make strong inferences about the associations between guppy local adaptation, diet, and seasonality.

Guppies are a very interesting model organism in evolutionary ecology, because the upstream and downstream populations have evolved different life-history traits in many different drainages as an effect of high or low predation pressure [21,41,46–48]. In the downstream sites, where predation is high, guppies grow faster, reproduce earlier and more frequently. They also produce smaller and more numerous offspring compared to the upstream sites where predation is low. Reznick [41] showed that guppy life history differences between HP and LP populations persist during the wet season but decrease in magnitude. For instance, during the wet season the size at maturity increases for both phenotypes, but in low predation sites increases more than in high predation. Fecundity and reproductive allotment, instead, decreases in the wet season, more so for guppies from high predation localities [41]. Contrary to the results for size at maturity, a decrease in these reproductive traits is consistent with the response to a decrease in food availability [49,50]. The greater decrease for these traits in high predation than low predation fish might indicate a bigger magnitude decrease in high quality resources (invertebrates) for high than for low predation sites. This trend is confirmed by our diet analysis, which shows a decline in invertebrates in the high predation fish diet but not in the low predation fish. Reznick [41] pointed out that the responses for size at maturity and reproduction were difficult to reconcile with one another on the basis of food availability alone, but they might be if both quantity and quality of food changed in a predictable fashion. In order to clarify these points, further studies should address the effect of seasonality and resources available to guppies’ diet to their life history traits.

### Gut length

We expected gut length to mirror the patterns found in the diet, so that nutrient absorption could be optimized [11,15]. That is, shorter guts would correspond to a more carnivorous diet (more invertebrates) and, vice versa, longer guts would correspond to a more herbivorous diet (more detritus and algae; [14,40]). We found such a correlation between the HP and LP sites in the Aripo River during the dry season. The large difference in diet (Fig 1A–Aripo HP vs LP during the dry season) was matched by the biggest difference in gut length among all of our samples (Fig 3A). In both drainages the percentage of invertebrates in the diet for high predation fish was around 70%. In the Aripo River the percentage of invertebrates in the diet in guppies from LP sites was ~10%, but it was up to ~40% for guppies in the Guanapo LP site. A similar but opposite pattern was found for the detritus. In spite of the reduced difference in diet during the wet season, HP guppies still maintained shorter guts than LP guppies, indicating that local adaptation might have a stronger effect on this trait than the environmental variability and changes in diet. Digestive tissues are costly to maintain and build [15], thus organisms could change their morphology only when there is a significant energy gain in doing so [51]. It might not be advantageous for guppies to change their morphology if changes in diet are not big enough and if they do not last for very long periods of time. Sullam and colleagues [19] showed similar results in a 10-weeks long controlled study, where HP and LP guppies were fed similar diets (high and low quality). They found that guppies’ gut length followed the changes in diet (poorer diets had longer guts), but the population of origin (HP or LP) had a stronger effect on gut length, maintaining differences in gut length in spite of the change in diet (HP always presented shorter guts than LP guppies).

In a study on 21 species of fish from Panama, Kramer and Bryant [40] showed that relative gut length declined progressively in comparisons among herbivorous, omnivorous, and carnivorous fish. However, within omnivores, they did not find differences in gut length between species consuming different proportion of plant material. Our data on the Aripo dry season differs from Kramer and Bryant’s [40] results, since we found that an increase in the proportion of invertebrates did appear to cause a reduction in gut length. Perhaps the absence of a significant effect of diet on gut length in the Guanapo River populations is attributable to the smaller difference between the high and low predation populations in the composition of the diet. Further work is required on the effect of local adaptation versus phenotypic flexibility in response to small changes in the relative proportion of animal vs vegetal matter in diet to fully characterize the relationship between diet and gut morphology in omnivorous species. Additionally, while we assume that seasonal and dietary differences in gut length are due to within individual reversible changes in gut length, i.e. phenotypic flexibility [8], we recognize that other factors may explain observed patterns in gut length. First, fixed developmental changes in response to early diet, i.e. developmental plasticity, may also occur. Little is known about whether early diet can have long-term effects on digestive traits (i.e. efficiency: [52]), but we cannot at present discount that such early effects may explain some of the variation in gut length we observed. Secondly, seasonal patterns in gut length may also arise not because of any flexibility but rather due to differential selection whereby individuals with longer guts are favored during the wet season while shorter guts are more advantageous during the dry season.

There was a positive allometric relationship between fish length and gut length, indicating that gut length increases faster than fish length. This result suggests that guppies might switch their diets with age. However, we found only limited evidence of an ontogenetic shift in diet in our gut content analysis. Only in the wet season was there a significant relationship between the proportion of invertebrates in diet and guppy length in both HP and LP populations, indicating that small fish are more carnivorous than bigger ones, which has been shown in other species of fish [53]. Nevertheless, considered that in the dry season we did not find evidence of an ontogenetic diet switch and that in the wet season it was a limited phenomenon (the regression equation only explained 15% of the variation), it is more likely that the allometry of gut length in guppies was explained with the necessity of maintaining the surface-to-volume ratio with increasing size [39]. An interesting and persistent feature of the differences in allometry between HP and LP populations is that the LP populations consistently have steeper slopes, meaning that the rate of increase in gut length with size is higher in LP populations. This is as consistent a feature of the differences between LP and HP fish as is the overall longer guts of LP guppies.

## Conclusions and Final Remarks

While our findings confirmed that guppies are omnivores [27], they also indicated that guppies have a broad range of variation in the proportion of invertebrates and detritus in their diet, which changed with season and was associated to local adaptation. These variations in diet were correlated with the gut length (Fig 6): guppies that showed higher levels of carnivory also had the shortest guts, and vice versa, those with higher levels of herbivory had longer guts. The flexibility of the digestive system may be an important adaptation that enables guppies to respond favorably to changes in food sources and maximize nutrient absorption and energy extraction from different food types. But gut length seemed also to have an evolutionary control (Fig 4), as the HP and LP sites still maintained differences in their gut length even when their diets become similar. For future studies we recommend including more replicates from HP and LP populations collected multiple times throughout seasons to understand the role of local adaptation in controlling gut length in guppies and to be able to understand to what extent gut length is plastic or controlled by evolutionary factors. Comparisons of gut length in F2 generation guppies under common garden conditions would also allow us to understand the genetic components of the differences we observed in the wild.

## Supporting Information

S1 Data Supporting Information 1. (XLSX)

S2 Data Supporting Information 2. (XLSX)
